# The Impact of a Dedicated Multidisciplinary Team Approach for Prosthetic Joint Infections of the Lower Limb

**DOI:** 10.1007/s43465-023-00842-5

**Published:** 2023-02-19

**Authors:** James D. Sires, Kim Pham, Christopher J. Wilson

**Affiliations:** 1 College of Medicine and Public Health, Flinders University, Adelaide, Australia; 2 Department of Orthopaedic Surgery, Flinders Medical Centre, Adelaide, Australia

**Keywords:** Prosthetic joint infection, Revision arthroplasty, Multidisciplinary team, Patient outcomes

## Abstract

**Introduction:**

Prosthetic joint infections (PJI) of the hip and knee have significant morbidity and mortality, and present with varying local, host and microbiological factors. Given the broad presentation and complexity of PJI’s, we developed a dedicated multidisciplinary team (MDT) to manage this complex patient cohort, and report on our early outcomes.

**Methods:**

This was a retrospective observational study of all patients diagnosed with a prosthetic joint infection of the hip or knee (*n = *71) at our institution during a 4.5-year period. Patients treated after development of the MDT (post-MDT) (*n = *44), were compared to a control group prior establishment of the MDT (pre-MDT) (*n = *27).

**Results:**

85.2% of individuals in the pre-MDT, and 85.7% of individuals in the post-MDT group were considered cured at a minimum 2 years post-operatively according to the Delphi-based definition. The total number of admissions to hospital (2.44 vs. 1.84) and total number of antibiotics used (3.37 vs. 2.75) decreased in the post-MDT group; however, differences were not considered statistically significant.

**Discussion:**

Implementation of a dedicated MDT in the management of individuals with PJI’s of the lower limb at our hospital has allowed early and effective collaboration between healthcare personnel, with early promising results. Given the broad nature of PJI, future studies are ongoing to determine modifiable risk factors to reduce the incidence and improve outcomes of individuals with PJI’s where systems can then be implemented into already established MDTs to achieve the best clinical outcome for our patients.

**Supplementary Information:**

The online version contains supplementary material available at 10.1007/s43465-023-00842-5.

## Introduction

The rate of joint arthroplasty continues to grow exponentially with Australia’s ever- increasing ageing population. The Australian Orthopaedic Association National Joint Replacement Registry reported almost 120,000 cases of joint replacement procedures performed in 2020 [1]. Based on recent growth, the incidence of total hip replacement (THR) and total knee replacement (TKR) is projected to increase by 208% and 276%, respectively, by 2030 [2]. Prosthetic joint infection (PJI) is one of the most devastating complications following total joint arthroplasty occurring in approximately 1–2% of cases, with rates often being slightly higher in those undergoing total knee arthroplasty [3].

PJI poses significant burden for the individual patient, the treating clinician, and the health care industry. Patients diagnosed with PJI are at an increased risk for significant morbidity, prolonged hospital stay, multiple surgeries, prolonged antimicrobial courses, loss of limb if requiring amputation, and even death [4–6]. Additionally, management of PJI is complex, depending on a multitude of local, host and microbiological factors including chronicity of infection, causative organisms, implant condition, soft tissue quality and patient comorbidities [4–8]. Treatment options include both medical and surgical strategies, ranging from washout and debridement of the joint, debridement and implant retention (DAIR) with exchange of removeable implants, 1-stage revision arthroplasty, 2-stage revision arthroplasty, arthrodesis, amputation, or treatment and/or suppression with antimicrobials alone [9, 10].

There has been extensive research into the development of classification systems, as well as treatment algorithms for the management of PJI [11, 12]; however, it can still be challenging for an individual surgeon to make treatment decisions alone as they often only see a small number of cases each year. This is compounded by the broad nature of presentation of PJI [13]. One method to address this is utilising a multidisciplinary team (MDT) approach which can assist in both diagnosis and management. Improved outcomes in other challenging fields such as oncology and polytrauma have been demonstrated when patients are managed by a collaborative team rather than by single individuals [14]. Although extensive work has been put into the development of models for MDT care of PJI and their implementation, there is still a paucity of research on the impact of a dedicated multidisciplinary team approach on the management of individuals with PJI.

At our arthroplasty unit, prior to 2017 PJIs were typically managed by the orthopaedic team with consults to other disciplines as needed. In October 2017, a monthly PJI MDT meeting was established involving orthopaedic surgeons who specialize in revision arthroplasty, infectious disease and microbiology physicians with significant musculoskeletal infection experience, microbiology registrars, orthopaedic fellows, residents, physiotherapists, a nurse consultant, and ad- hoc input from plastic surgeons, vascular surgeons and dieticians (Appendix 1 in supplementary material). This meeting involves a collaborative discussion between all members to discuss individual cases and determine both a diagnosis and appropriate treatment plan based on wholistic needs of the patient.

It is postulated that following the implantation of our MDT approach to management of PJI, 2-year outcomes for patients diagnosed with PJI would improve. The aim of this study was to compare patient outcomes before and after the implementation of our arthroplasty unit’s MDT meeting.

## Methods

This was a single institution, retrospective review of individuals with a PJI of the hip or knee managed by the arthroplasty unit between October 2015 and April 2020. PJI cases were identified via the arthroplasty unit’s electronic audit software. To assess the impact of the MDT, we compared a control group prior to the establishment of the MDT meeting labelled pre-MDT which involved cases between September 2015 to September 2017, to a treatment group labelled post-MDT which included cases between October 2017 to April 2020. Individuals were included if at time of review their diagnosis of PJI was confirmed with 2018 MSIS criteria [7] and had a minimum 2-year follow-up from initial review. Exclusion criteria included patients reviewed at MDT for diagnosis other than PJI such as native septic arthritis, complex metalware infections not involving the joint, and individuals thought to have had a PJI but subsequently were considered not too.

### Data Collection

Patient demographics, body mass index (BMI), past medical history, American Society of Anaesthesiologist (ASA) grade, laboratory investigations, microbiological profile, antibiotic treatment, and surgical management were recorded. Outcome data recorded included total admissions to hospital, total length of stay (LOS), number of surgeries, number of antibiotics used, mortality (all-cause) and mortality (related to PJI). Additionally, the Delphi-based criteria [15] which included the following: (1) infection eradication, characterized by a healed wound without fistula, drainage, or pain, and no infection recurrence caused by the same organism strain; (2) no subsequent surgical intervention for infection after reimplantation surgery; and (3) no occurrence of PJI-related mortality was also recorded. Individuals meeting all these criteria at 2 years post-operatively were considered cured.

### Analysis and Outcomes

Data was analysed using STATA BE version 17. Means and standard deviations were calculated for numerical data, and proportions were calculated and compared for categorical data. Statistical tests utilized were the 2-sample *t*-test to compare numerical data, and the difference in 2 proportions test for categorical data.

Ethics review was not sought because the study met criteria for exemption from such review according to an institutional policy.

## Results

A total of 71 individuals with a confirmed prosthetic joint infection were treated and managed over a 4.5-year period, with 44 individuals in the post-MDT group. Follow-up of patients ranged from 2.22 to 6.72 years. Overall, mean age of patients was 72.32 (11.62) years, with 42 (59.15%) being female. Mean BMI was 31.90 (6.55) kg/m^2^, and mean ASA score was 2.64 (0.77). More than half of individuals (60.56%) were diagnosed with a chronic prosthetic joint infection. Further details on demographics, past medical history and type of prosthetic joint infection are shown in Table 1. There were no statistically significant differences in these demographics between the two groups.

**Table 1:** Demographics, past medical history and prosthetic joint infection characteristics of individuals managed pre and post-MDT

	Pre-MDT	Post-MDT	*p*
*N*	27	44	
Age (years)	72.82 (2.08)	72.02 (1.84)	0.78
Gender (F)	15 (55.6%)	27 (61.4%)	0.63
PMHx			
Diabetes	4 (14.8%)	7 (15.9%)	0.90
ASA	2.52 (0.80)	2.73 (0.76)	0.28
BMI (kg/m^2^)	31.15 (6.09)	32.45 (6.91)	0.44
Smoker	9 (33.3%)	12 (27.3%)	0.59
Albumin (mg/dL)	31.07 (6.11)	29.77 (5.94)	0.40
Knee involvement	17 (37.0%)	20 (45.5%)	0.49
Prior revision surgery	6 (22.2%)	9 (20.9%)	0.86
Chronic PJI	17 (63.0%)	26 (60.5%)	0.75

Microbiological profile of PJI’s is highlighted in Table 2. Most cases involved a Gram-positive organism (70.42%), and some contained a Gram-negative organism (35.21%). There were 2 cases involving a yeast (*Candida albicans*), and 2 cases involving a fungus (*Aspergillus niger*), both of which were in individuals in the post-MDT group. In total, 26.76% of cases involved 2 or more organisms (polymicrobial).

**Table 2:** Microbiological profile of prosthetic joint infections of individuals managed pre and post-MDT

	Pre-MDT (27)	Post-MDT (44)
Gram-positive organism	22 (81.5%)	28 (63.6%)
MSSA^a^	7	9
MRSA^b^	3	2
CoNS^c^	8	11
Other G +	8	12
Gram-negative organism	9 (33.3%)	16 (36.4%)
Pseudomonas	3	7
Other	7	10
Yeast (*Candida albicans*)	0 (0.0%)	2 (4.5%)
Fungus (*Aspergillus niger*)	0 (0.0%)	2 (4.5%)
Nil growth	1 (3.7%)	6 (13.6%)
Polymicrobial	7 (25.9%)	12 (27.3%)

^a^Methicillin-sensitive *Staphylococcus aureus* (MSSA)

^b^Methicillin-resistant *Staphylococcus aureus* (MRSA)

^c^Coagulase-negative Staphylococci (CoNS)

Surgical management predominantly consisted of a washout and debridement (28.17%), debridement-antibiotic and implant retention with exchange of exchangeable components (25.35%), or a 2-stage revision arthroplasty (38.03%). Only 3 individuals underwent a 1-stage revision, 2 underwent an amputation and 1 patient underwent an arthrodesis of their knee (Table 3).

**Table 3:** Surgical management of individuals managed pre and post-MDT

	Pre-MDT (27)	Post-MDT (44)
Washout and debridement	8 (29.6%)	12 (27.3%)
DAIR	3 (11.1%)	15 (34.1%)
One stage revision	2 (7.4%)	1 (2.3%)
Two stage revision (including intermittent antibiotic spacer)	13 (48.1%)	14 (31.8%)
Other (amputation/fusion)	1 (3.7%)	2 (4.5%)

In total, 85.2% of individuals in the pre-MDT, and 85.7% of individuals in the post-MDT group were considered cured at a minimum 2 years post-operatively according to the Delphi-based definition. Total admissions (2.44 vs 1.84), length of stay (43.44 days vs 42.79 days), and number of antibiotics used (3.37 vs 2.75) were reduced in the post-MDT group, however, results were not considered statistically significant (Table 4). Overall, all-cause mortality of the whole cohort was 14.08%.

**Table 4:** Outcomes of individuals managed pre and post-MDT

	Pre-MDT	Post-MDT	*p*
Delphi criteria > 2 years (include all-cause mortality)	23/27 (85.2%)	36/44 (81.8%)	0.71
Delphi Criteria > 2 years (exclude non PJI-related death)	23/27 (85.2%)	36/42 (85.7%)	0.95
Total admissions	2.44 (1.95)	1.84 (0.97)	0.09
Total LOS (days)	43.44 (38.09)	42.79 (28.44)	0.94
Number of surgeries	1.93 (1.33)	2.25 (1.69)	0.40
Number of antibiotics	3.37 (0.42)	2.75 (0.26)	0.19
Mortality (all-cause)	3 (11.1%)	7 (15.9%)	0.57
Mortality (PJI related)	3 (11.1%)	5 (11.4%)	0.97

## Discussion

### Summary

Implementation of a multidisciplinary team approach for the management of individuals with prosthetic joint infections of the lower limb resulted in a reduction in the length of stay, number of hospital admissions and total number of antibiotics used over the treatment period; however, these findings were not statistically significant.

### Comparison to Other Studies

Few studies have investigated the effects of the multidisciplinary approach on PJI management and patient outcomes. Karczewski et al. found the rate of recurrent infection (10.4 vs 3.1%) decreased considerably with the introduction of the interdisciplinary team for PJI management [16]. Furthermore, Vuorinenet al. describes implementation of an MDT resulted in a reduction in the median number of surgeries (2 vs 1), as well as decreased median length of stay (49 vs 17 days) in patients diagnosed with lower limb PJI [17]. Lastly, Ntalos et al. showed post-MDT implementation there was a lower usage of total antibiotics (4.2 vs 2.8), decreased LOS (62 vs 29 days) and a reduction in number of surgeries (5.1 vs 1.8) [18]. It is thought that a number of these factors are inter-linked, and potentially driven by the early and coordinated input of infectious disease and microbiology physicians at or near the time of diagnosis of a PJI. Often, individuals are prescribed long courses of antibiotics up to 6 months [19, 20], however, shorter courses of antibiotic therapy may be appropriate for most cases of PJI if specific organisms are targeted potentially resulting in a reduction in microbiological resistance and length of hospital stay [21–23]. Results of our study and Ntalos [18] could be attributed to having coordinated infectious disease specialists involved early in the management of PJI, driving improved antimicrobial stewardship compared to pre-MDT management where targeted and appropriate management could have been delayed, resulting in an increase in the number of antibiotics used in the interim, as well as an increase in length of stay.

Furthermore, in comparison to pre-MDT, patients managed post-MDT implementation had higher rates of DAIR and exchange of exchangeable components as the main surgical treatment (11.1 vs 34.1%) compared to washout and debridement alone (29.6 vs 27.3%). An increase in proportion of patients having DAIR as primary surgical procedure with MDT implementation was also demonstrated by Vuorinenet al. (42.9 vs 89.6%) [17]. Several studies have recommended DAIR as the preferred treatment option for acute PJI [24, 25] and that arthroscopic washout procedures should be considered as only temporizing procedures, predominantly indicated for patients who are acutely unwell and cannot tolerate a DAIR or revision arthroplasty [26]. Increased rates of DAIR within the post-MDT group could be a result of better adherence to clinical and up-to-date treatment recommendations by the involved clinicians in the MDT meeting. Furthermore, our results show a reduction in the number of hospital admissions in the post-MDT group, potentially because of the known possible increased effectiveness DAIR offers over washout and debridement [27]. Through providing best practice to patients with PJI, this may lessen the financial burden PJI’s have on the healthcare system through reducing total number of procedures and re-admissions for recurrent disease, as well as improve patient quality of life [28, 29]. A study conducted in 2013 by Peel et al. found the median cost of treating PJI in Australia was $34,800 per patient which included hospitalization costs and antibiotic therapy costs [30], and therefore reducing number of admissions and antibiotic use will theoretically lead to potential significant savings to the healthcare system.

Interestingly, while Vuorinen et al. [17] and Ntalos et al. [18] showed a reduction in surgeries in patients discussed in the MDT meeting, there was a slight increase in the number of surgeries performed within our post-MDT group; however, this was not statistically different. This could be potentially explained by difference in the microbiological profile distribution between the two groups. In total, two patients in the post-MDT group had fungal infections, and prior literature suggests these to be both difficult to diagnose and manage [31, 32]. In each case initial intraoperative cultures did not identify a fungal organism, however, once identified these were managed with first stage revision arthroplasty often needing repetition until the patient had both clinical and biochemical improvement prior proceeding to second stage revision arthroplasty. Furthermore, given these sub-types of PJI were uncommon at our centre these cases were discussed with international colleagues from the University of Oxford, United Kingdom. Differentiating aspects of treatment included utilisation of intravenous anti-fungal agents, topical anti-fungal use in cement spacer, and extended duration of antibiotics post second stage revision arthroplasty. One case in the post-MDT group involved a patient with a chronic PJI involving polymicrobial growth which included *Candida albicans*, *MRSA* and *Aspergillus niger*— all of which are known difficult-to-treat organisms [33]. This patient over a 2.5-year period underwent 8 operations given the number of recurrent episodes of PJI. Given PJI broad presentation, including whether they involve difficult-to-treat organisms, comparison does become difficult as outcome from set management may be confounded by a multitude of factors [33–35].

### Defining Treatment Success for PJI

The definition of treatment success for PJI is variable in the literature which results in limitations when comparing the results of available studies. Some investigators define treatment success as the absence of signs and symptoms suggestive of infection at a defined follow-up point [36, 37]. On the other hand, Laffer et al. [38] described patients “definitely free of infection” constituted: no signs of infection, CRP ≤ 10 mg/L and follow-up ≥ two years. Our study uses the success definition resulted from a Delphi-based multidisciplinary consensus by an expert panel [15] (Table 5).

**Table 5:** Definition of success for PJI treatment and its temporal classification [15]

Definition of a successfully treated PJI	Infection eradication, characterized by a healed wound without fistula, drainage, or pain, and no infection recurrence caused by the same organism strain
	No subsequent surgical intervention for infection after reimplantation surgery
	No occurrence of PJI-related mortality (by causes such as sepsis, necrotizing fasciitis)
Temporal classification of success	Short-term: 2 years
	Midterm: 5–10 years
	Long term: 10 years or more

Based on this definition, no significant difference could be found comparing the success of management in achieving cure between pre- and post-MDT groups in our study, of which approximately 85% of patients in each group met the Delphi-based definition at 2-years follow-up. Multiple factors may have contributed to this, including the varying microbiological profiles and PJI type between each group. Other studies reporting treatment success include Davis et al. [39], who analysed 2-year outcome data of patients with large joint PJI and reported on clinical cure (defined as: alive, absence of clinical or microbiological evidence of infection, and not requiring ongoing antibiotic therapy), and treatment success which was defined as clinical cure (as above) plus index prosthesis still in situ. Based on these definitions, clinical cure rate was 69%, while 54% of patients had treatment success in this study, which appear quite low however this could be contributed to by how they variably defined treatment success.

### Limitations

We acknowledge several limitations exist in our study. Firstly, the design was retrospective and observational, with groups determined based on time-periods and not randomization potentially introducing other time-based confounders into the study. Additionally, individuals diagnosed with a PJI have variable local, host and microbiological factors, and given the relatively small sample size, inhomogeneity is highly likely to exist. For example, the post-MDT group had patients who had a PJI involving a fungus/yeast which are known difficult-to-treat organisms potentially impacting on the overall groups outcome [31, 32]. Furthermore, since establishment of our MDT we have actively encouraged surgeons in other centres in our region to refer cases for ongoing management, especially ones considered more ‘difficult’, potentially introducing a bias towards the more difficult-to-treat PJI’s. Lastly, the impact of introducing an MDT approach to institution is site specific, as outcomes would depend on both the available resources and personnel that compromise the MDT, and therefore implementing such a system/approach in an alternate setting may have varying results.

## Conclusion

Implementation of an MDT approach in management of individuals with PJI’s at our hospital has allowed early and effective collaboration of infectious disease, microbiology, and allied healthcare workers with orthopaedic surgeons. Given the broad nature of PJI, future studies are ongoing to determine modifiable risk factors to reduce the incidence and improve outcomes of individuals with PJI where systems can then be implemented into already established MDTs to achieve the best clinical outcome for our patients.

## Supplementary Information

Below is the link to the electronic supplementary material.

Supplementary file1 (DOCX 30 kb)

## Data Availability

Authors can confirm that all relevant data are included in the article and/or its supplementary information files.
