# Supplementary material for: The Impact of a Dedicated Multidisciplinary Team Approach for Prosthetic Joint Infections of the Lower Limb
Source: Indian J Orthop. 2023 Feb 19;57(5):696–702. doi: 10.1007/s43465-023-00842-5 (PMC10147879; doi:10.1007/s43465-023-00842-5)
Supplement: Supplementary file 1 — Supplementary file1 (DOCX 30 kb) [file 43465_2023_842_MOESM1_ESM.docx]

# Appendix

| **Team Member** | **Role** |
| --- | --- |
| **Orthopaedic Surgeons**  **(Revision arthroplasty specialists)** | Provide revision arthroplasty specialist input. Discuss surgical management options available, highlighting difficulties/potential challenges based on patient anatomy, such as bone stock, and hardware/implant factors. |
| **Orthopaedic Fellow/Registrars** | Review and admit patients to hospital when present through emergency department. Discuss and present cases to MDT meeting. Review clinical progress and response to treatment. |
| **Infectious Disease Physicians**  **(Musculoskeletal infection specialists)** | Provide specialist musculoskeletal infectious disease input. Discuss and review plan for intra-operative sample investigations, advice on antibiotic commencement, selection, and duration. Provide advice on antibiotic selection in cases where use is topical (powder, within cement spacers, beads). Advise on further investigation/work-up to causes of PJI (haematological spread). |
| **Microbiology Registrars** | Review and discuss acutely unwell patients admitted through emergency department in liaison with orthopaedic team. Discuss and review available microbiology results, providing advice on antibiotic selection. Review and monitor biochemical and microbiological results for changes/updates. |
| **Nurse Consultants** | Coordination between team members. Patient liaison, pre and post operative coordination of clinic appointments and theatre bookings. Often first line if patient calls hospital with symptoms of an acute PJI, prompting urgent review via emergency department or in clinic. |
| **Dieticians** | Pre-operative, or during admission nutritional screening, assessment and supplementation were indicated. |
| **Physiotherapists** | Pre-operative assessment and discharge planning in chronic cases. Inpatient and post-operative rehabilitation. |
| **Adhoc Input** | The MDT meeting has several regular attendees, however depending on the cases discussed, additional input/visitation occurs from plastic and reconstructive surgeons, vascular surgeons, and anaesthetists.  Furthermore, challenging/complex cases are discussed with international colleagues at University of Oxford, United Kingdom for advice, such as those with fungal infections. |
